# PCB defect detection algorithm based on CDI-YOLO

**DOI:** 10.1038/s41598-024-57491-3

**Published:** 2024-03-28

**Authors:** Gaoshang Xiao, Shuling Hou, Huiying Zhou

**Affiliations:** School of Computer and Information Engineering, Central South University of Forestry and Technology, Changsha, 410004 China

**Keywords:** Engineering, Electrical and electronic engineering

## Abstract

During the manufacturing process of printed circuit boards (PCBs), quality defects can occur, which can affect the performance and reliability of PCBs. Existing deep learning-based PCB defect detection methods are difficult to simultaneously achieve the goals of high detection accuracy, fast detection speed, and small number of parameters. Therefore, this paper proposes a PCB defect detection algorithm based on CDI-YOLO. Firstly, the coordinate attention mechanism (CA) is introduced to improve the backbone and neck network of YOLOv7-tiny, enhance the feature extraction capability of the model, and thus improve the accuracy of model detection. Secondly, DSConv is used to replace part of the common convolution in YOLOv7-tiny to achieve lower computing costs and faster detection speed. Finally, Inner-CIoU is used as the bounding box regression loss function of CDI-YOLO to speed up the bounding box regression process. The experimental results show that the method achieves 98.3% mAP on the PCB defect dataset, the detection speed is 128 frames per second (FPS), the parameters is 5.8 M, and the giga floating-point operations per second (GFLOPs) is 12.6 G. Compared with the existing methods, the comprehensive performance of this method has advantages.

## Introduction

As a basic component of the vast majority of electronic devices, PCBs play the role of a skeleton for connecting electronic components as well as for transmitting signals and energy. The manufacturing process of PCB, needs to go through several complex processes, each of which may have quality defects, which can be divided into two categories: functional defects and appearance defects^1^. functional defects are the most serious during PCB production and can directly affect the performance of the PCB, while appearance defects can affect the aesthetics. In these two types of defects, six main types of defects often occur in industrial production, including missing hole, mouse bite, open circuit, short, spur, and spurious copper.

In early industrial production, PCB defect detection methods are mainly based on manual visual inspection, functional inspection, and inline instrumentation. However, the manual visual inspection method has the disadvantages of visual fatigue, slow detection speed, and high cost; the functional inspection method requires special test equipment and cannot detect multiple defects; and the inline inspection method can only detect functional defects. Currently, automated optical inspection (AOI)^2^ has become the most common PCB defect detection technology used in industrial production. AOI is a non-contact inspection method based on machine learning and image processing technology. There are three basic methods of AOI, which are the reference comparison method, non-reference comparison method, and Hybrid method^3^. The reference comparison method is used to determine the type of defect by comparing the difference between the PCB to be inspected and the PCB stencil, but this method is more affected by external influences such as lighting. The non-reference comparison method requires predesigned discrimination conditions, but the presence of a defect is not set in advance of the discrimination conditions can not detect the defect. The hybrid method is a combination of the first two methods, but the combination of the algorithm increases the amount of calculation, and the detection steps are cumbersome.

In recent years, deep learning methods have gradually dominated the image field, and deep learning algorithms are highly accurate and fast compared to machine learning. Deep learning-based object detection algorithms are mainly classified into two main categories, end-to-end One-stage object detection algorithms, such as YOLOv1^4^, YOLOv2^5^, YOLOv3^6^, YOLOv4^7^, and SSD^8^; and Two-stage object detection algorithms based on region suggestions, such as R-CNN^9^, Fast R-CNN^10^, and Faster R-CNN^11^. Deep learning algorithms are widely used in the field of defect detection. Yanan et al.^12^ used the YOLOv3 algorithm to achieve surface defect detection of steel rails. Wang et al.^13^ used deep learning neural network for defect detection on potato surface. Zhang et al.^14^ proposed a solar surface defect detection algorithm by fusing multi-channel convolutional neural network. Some scholars have also applied deep learning methods in the field of PCB defect detection. Ding et al.^15^ proposed TDD-net based on Faster R-CNN, which used the k-means clustering method to obtain more reasonable anchor frames and enhanced feature mapping relationships at different levels, suitable for the detection of small defects. Hu et al.^16^ constructed a new network based on improved Faster R-CNN, which used ResNet50 as the backbone network, used GARPN to predict more accurate anchor frames, and merged the residual units of Shufflenetv2. This method does not require external mechanical fixtures and strict template alignment operations, reducing the testing cost. Chen et al.^17^ proposed a Transformer-YOLO network detection model using an improved clustering algorithm to generate suitable anchor frames, using Swin Transformer as a feature extraction network, and adding convolution and attention mechanism modules to the feature detection network in order to make the network more effective in focusing on defect information. Liu et al.^18^ proposed a fast defect detection network, which used a modified MobileNetv2 network as the backbone network, introduced the SPP structure, and used k-means clustering to obtain a suitable anchor frame, and the experimental results showed that the network model was small and could be detected in real-time. Liao et al.^19^ improved the YOLOv4 network by using a lightweight network MobileNetv3 replaced the CSPDarknet53 backbone network of YOLOv4 with an optimized activation function, and the experimental results showed that the network was improved in both detection accuracy and speed and reduced the number of model parameters.

However, the current deep learning-based method for PCB defect detection does not balance detection accuracy, speed, and network model parameters well. To address this issue, this paper proposes a new network model, CDI-YOLO, by selecting the lightweight YOLOv7-tiny network model as the baseline and making relevant improvements. The paper’s main contributions are:

- (1) The YOLOv7-tiny network model combines the ELAN structure with the CA module. The CA module allows the network model to consider both the relationship between feature channels and the location information in the feature space during training. This is beneficial for the network model to focus more on the PCB defective features during feature extraction, ultimately improving the detection accuracy of the network.
- (2) DSConv can be used to replace some of the common convolutions in the YOLOv7-tiny network model. This technique mimics the behavior of convolutional layers by using quantization and distributional offsets, resulting in lower computational effort and faster detection.
- (3) CDI-YOLO uses Inner-CIoU as the bounding box regression loss function instead of CIoU. Inner-CIoU generates auxiliary bounding boxes of different sizes using scale factors to compute the loss values, resulting in faster and more efficient regression results.

## Methodology

### Overview of the YOLOv7-tiny network model

**Figure 1:**
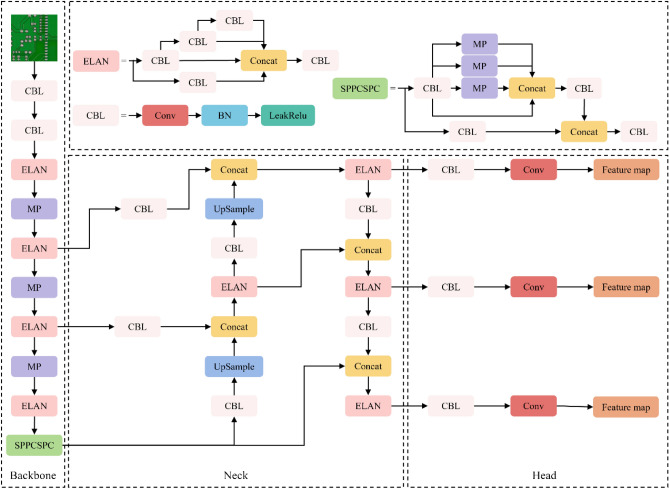
YOLOv7-tiny network structure.

In 2022, Wang et al.^20^ proposed the YOLOv7 object detection algorithm. The algorithm includes models with different widths and depths for edge GPUs, normal GPUs, and cloud GPUs, such as YOLOv7-tiny, YOLOv7, and YOLOv7-W6. Scaling strategies are used to generate YOLOv7-X, YOLOv7-E6, and other new models. To achieve a balance between detection accuracy, speed, and network model parameters in the PCB defect detection task, this paper utilizes the YOLOv7-tiny network model, a lightweight version of the YOLOv7 network model, as the baseline model. Relevant improvements are made based on this model. Figure 1 shows the structure diagram of the YOLOv7-tiny model.

The YOLOv7-tiny network model’s basic framework comprises four main components: Input, Backbone, Neck, and Head.

The input layer utilizes Mosaic data augmentation to randomly crop input images before splicing them into a single image for training data, thereby enriching the dataset.

The backbone network comprises CBL, ELAN, SPPCSPC, and MP modules. The CBL module includes a convolutional layer, a normalization layer, and a LeakRelu activation function. The ELAN module consists of multiple CBL modules, and the MP is a maximum pooling layer. The backbone network extracts features from the image.

The PANet (Path Aggregation Network)^21^ construct is used by Neck as its feature fusion module. This allows for information aggregation through top-down and bottom-up paths, enabling features at different scales to communicate and fuse with each other, thereby improving the accuracy of object detection.

The Head of YOLOv7 adopts the feature pyramid structure commonly used in the YOLO series, where different levels of feature maps are processed and fused to capture object information at different scales. The detection head comprises several prediction layers that forecast the object’s bounding box, category, and confidence score.

### CDI-YOLO network structure

**Figure 2:**
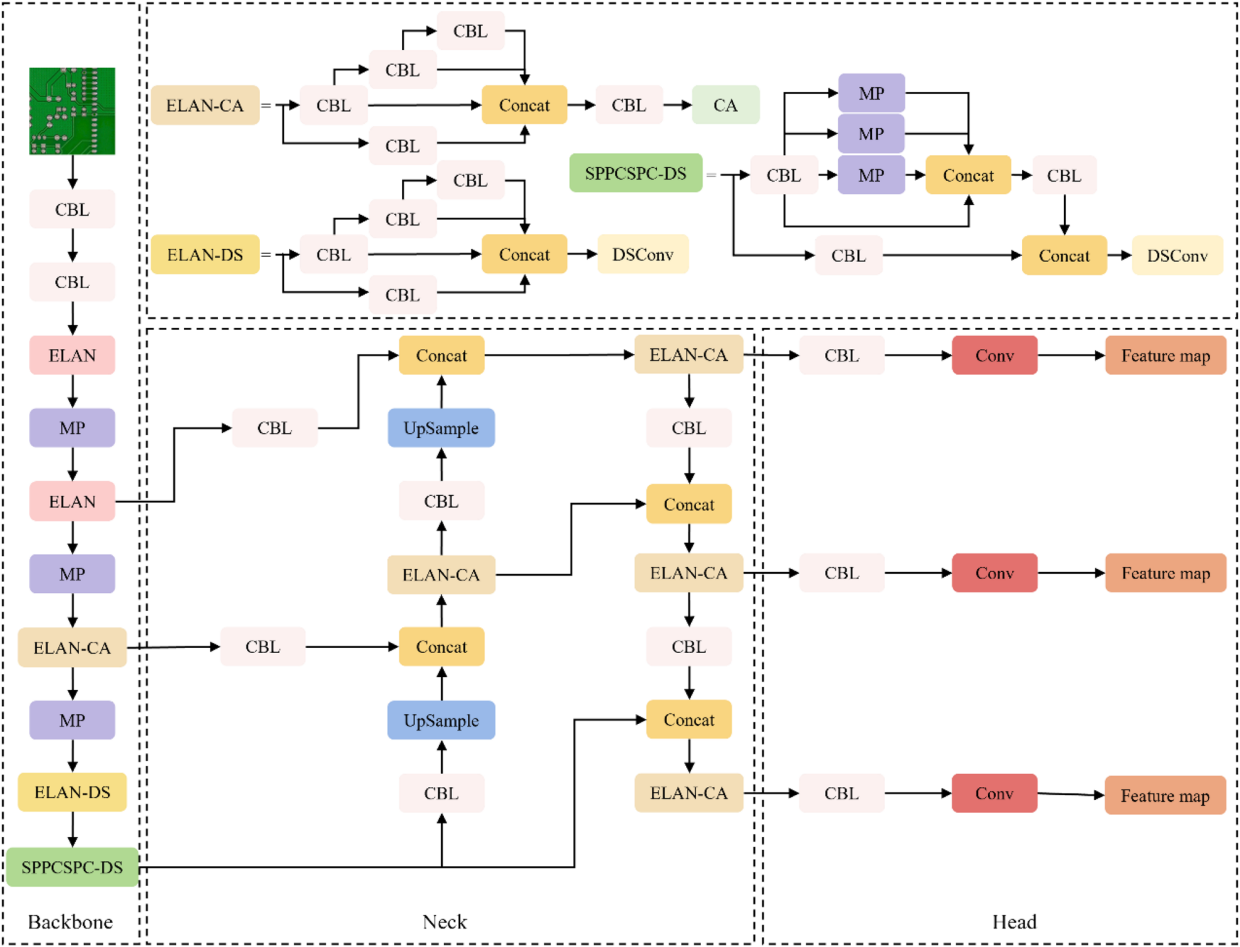
CDI-YOLO network structure.

The CDI-YOLO network model is an improvement of YOLOv7-tiny. The model structure is shown in Fig. 2. The CA module is combined with the ELAN structure^22^ of YOLOv7-tiny and embedded into the backbone and neck networks of the network model. This enables the model to focus globally on various locations of the inputs instead of limiting itself to specific regions, improving the accuracy of network detection. DSConv was used to replace some of the CBL modules in the YOLOv7-tiny network model to improve its ability to detect various types of PCB defects. Additionally, Inner-CIoU was employed as the bounding box regression loss function for CDI-YOLO, resulting in faster bounding box regression through the use of an auxiliary bounding box to calculate the loss.

#### Introduction of coordinated attention module

Due to the small defects of PCBs, feature information may not be immediately apparent and can be influenced by various environmental factors. To enhance the YOLOv7-tiny network model’s ability to extract defect information from PCBs, this study incorporates the CA module into the ELAN module of YOLOv7-tiny^23^. This improves the accuracy of PCB defect detection.

**Figure 3:**
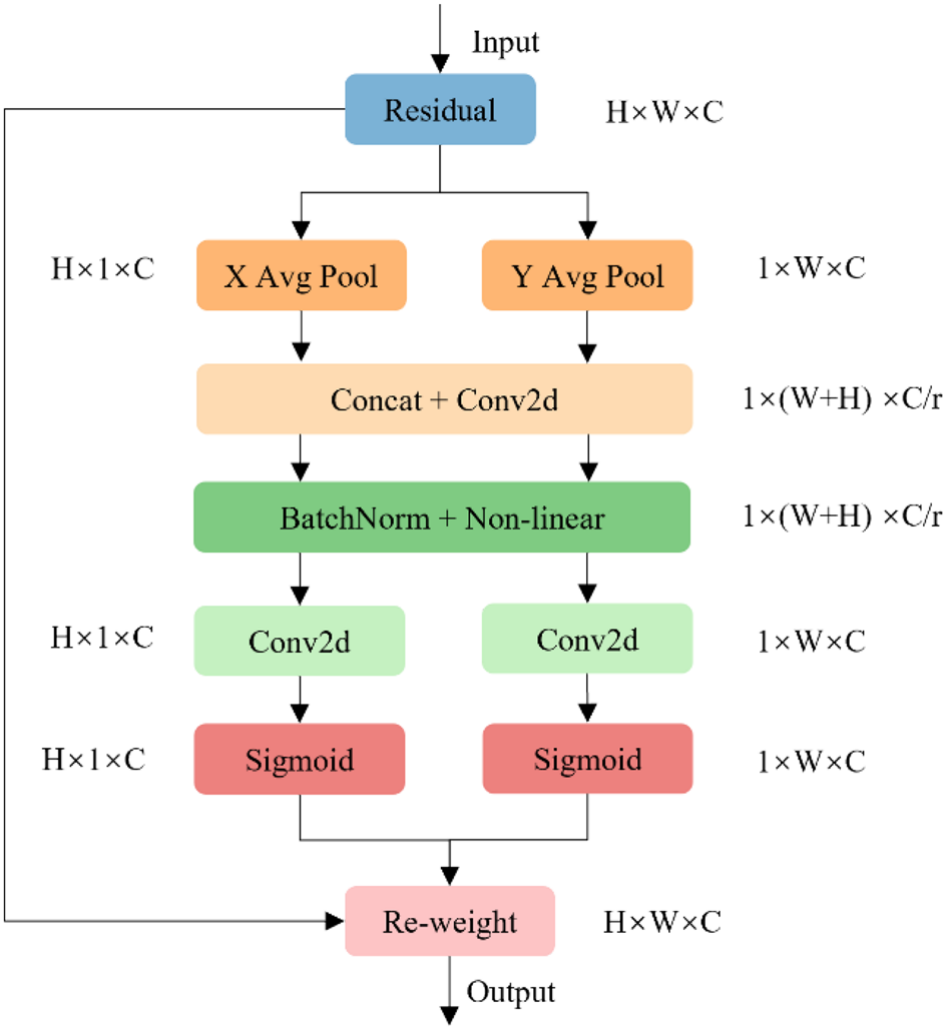
CA module.

CA module is an attention mechanism used in computer vision tasks to improve model performance by enhancing feature representation. Traditional attention mechanisms focus on the channel dimensions of the feature map, dynamically adjusting the feature importance between channels by learning weights. Conversely, CA module concentrates on the spatial location of the feature graph and adjusts the importance of different spatial locations by learning their weights. The fundamental concept of CA module is to incorporate the location information of the feature graph into the attention weights. The approach takes into account that features located in different areas may have varying contributions to the task. As a result, it adjusts the importance of features by learning the weights of their locations to better capture spatially structured information. The CA module encodes channel relationships and remote dependencies through two steps: coordinate information embedding and coordinate attention generation. Figure 3 illustrates the coordinate attention module, and the detailed principle of CA module is described below.

(1) Coordinate Information Embedding.

The input feature map $X$ undergoes pooling operations along the horizontal and vertical directions using two pooling kernels, $(H,1)$ and $(1,W)$, respectively. Equation (1) shows the output of channel c in the vertical direction h.

1 $${z}_{c}^{h}\left(h\right)=\frac{1}{W}\sum_{0\le i<W}{x}_{c}\left(h,i\right)$$

Equation (2) shows the output of channel c in the horizontal direction w.

2 $${z}_{c}^{w}\left(w\right)=\frac{1}{H}\sum_{0\le j<H}{x}_{c}\left(j,w\right)$$

The horizontal and vertical outputs are then spliced to obtain a pair of orientation-aware feature maps $Z$.

(2) Coordinate Attention Generation.

Equation (3) shows that the feature map $Z$, obtained through coordinate information embedding, is input into a 1 × 1 convolutional kernel ${F}_{1}$, followed by a nonlinear activation operation $\delta$.

3 $$f=\delta \left({F}_{1}\left(Z\right)\right)$$

The feature map $f$, obtained by Eq. (3), is split into two tensors: ${f}^{h}\in {R}^{C/r\times H}$ and ${f}^{w}\in {R}^{C/r\times W}$, along the horizontal and vertical directions, respectively. Following this, two 1 × 1 convolution kernels, ${F}_{h}$ and ${F}_{w}$, are used to convert ${f}^{h}$ and ${f}^{w}$ into tensors ${g}^{h}$ and ${g}^{w}$, respectively, with the same number of channels as the input $X$. These are computed as shown in Eq. (4) and Eqs. (5).

4 $${g}^{h}=\sigma \left({F}_{h}\left({f}^{h}\right)\right)$$

5 $${g}^{w}=\sigma \left({F}_{w}\left({f}^{w}\right)\right)$$

where $\sigma$ is the sigmoid activation function. Then the outputs ${g}^{h}$ and ${g}^{w}$ from Eq. (4) and Eq. (5) are multiplied as weights with the initially input feature map $X$. Finally, the output $Y$ of the coordinate attention module is shown in Eq. (6).

6 $${y}_{c}\left(i,j\right)={x}_{c}\left(i,j\right)\times {g}_{c}^{h}\left(i\right)\times {g}_{c}^{w}\left(j\right)$$

Through these steps, CA module can adjust feature weights based on location importance, improving the model’s ability to capture spatially structured information. This mechanism enhances the model’s accuracy in perceiving and understanding important spatial locations in computer vision tasks.

#### DSConv module

**Figure 4:**
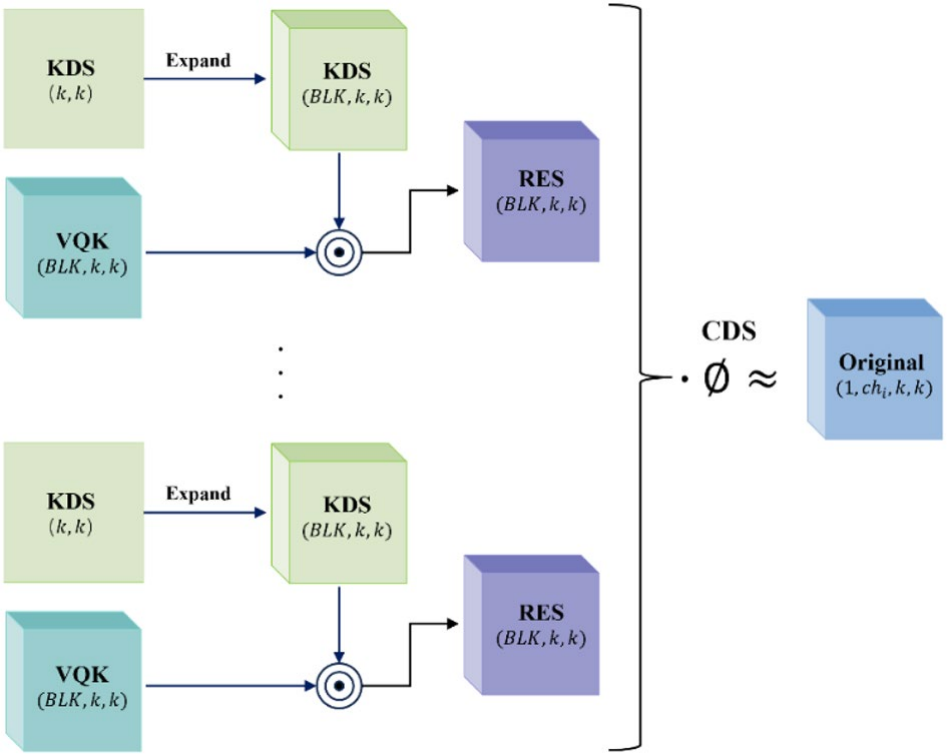
Basic principle of DSConv.

DSConv^24^ is a variant of the traditional convolutional layer. By replacing the ordinary convolution with DSConv, it is possible to achieve lower computation and higher detection speed. The principle of DSConv is shown in Fig. 4.

DSConv decomposes the operation of a traditional convolutional layer into Variable Quantized Kernel (VQK) and Distribution Shifts. VQK is the quantised component of DSConv with the same size $({ch}_{o},{ch}_{i},k,k)$ as the original convolution tensor. Here, ${ch}_{o}$ denotes the number of output channels, ${ch}_{i}$ denotes the number of input channels, and $k$ denotes the size of the convolution kernel. The parameter values are obtained by quantising the original floating-point model into variable bit-length integer values. Once the parameter values have been quantised, they cannot be changed. Distribution shifts are used to adjust the distribution of the VQK by two tensors: the Kernel Distribution Shifter (KDS) and the Channel Distribution Shifter (CDS). The KDS are used to carry out distribution shifts on each $(1,BLK,\mathrm{1,1})$ slice of the VQK, on which the distribution is shifted. BLK is a hyperparameter that determines the block size for the VQK depth values in each displacement operation. Each value in the KDS corresponds to a displacement operation that shifts BLK depth values of the VQK. The size of the KDS is $2 \cdot \left( {ch_{o} ,CEIL\left( {\frac{{ch_{i} }}{BLK}} \right),k,k} \right)$ where $CEIL(x)$ is an upward rounding operator used to ensure that the computed dimensions satisfy the requirements. The size of CDS is $2 \cdot \left( {ch_{o} } \right)$. The CDS distributes the displacements on each channel by performing a distributed displacement operation on each $(1,{ch}_{i},k,k)$ slice.

#### Inner-CIoU loss

The YOLOv7-tiny model employs the CIoU bounding box regression loss function. However, this function has the disadvantage of slow convergence. To address this issue, we use the Inner-CIoU loss^25^ as the bounding box regression loss function.

The Inner-CIoU loss calculates the loss based on the CIoU loss using an auxiliary bounding box, which is defined as shown in Eq. (7).

7 $${L}_{inner-CIoU}={L}_{CIoU}+IoU-{IoU}^{inner}$$

where ${L}_{CIoU}$ denotes the CIoU loss function, $IoU$ denotes the intersection and concatenation ratio of the predicted and real frames, and ${IoU}^{inner}$ is defined as shown in Eq. (8).

8 $${IoU}^{inner}=\frac{inter}{union}$$

The definitions of $inter$ and $union$ are shown in Eq. (9) and Eqs. (10):

9 $$inter=\left(min\left({b}_{r}^{gt},{b}_{r}\right)-max\left({b}_{l}^{gt},{b}_{l}\right)\right)*\left(min\left({b}_{b}^{gt},{b}_{b}\right)-max\left({b}_{t}^{gt},{b}_{t}\right)\right)$$

10 $$union=\left({w}^{gt}*{h}^{gt}\right)*{\left(ratio\right)}^{2}+\left(w*h\right)*{\left(ratio\right)}^{2}-inter$$

where ${b}_{r}^{gt}$, ${b}_{r}$, ${b}_{l}^{gt}$, ${b}_{l}$, ${b}_{b}^{gt}$, ${b}_{b}$, ${b}_{t}^{gt}$ and ${b}_{t}$ are defined as follows.

11 $${b}_{r}^{gt}={x}_{c}^{gt}-\frac{{w}^{gt}*ratio}{2},{b}_{r}^{gt}={x}_{c}^{gt}-\frac{{w}^{gt}*ratio}{2}$$

12 $${b}_{t}^{gt}={y}_{c}^{gt}-\frac{{h}^{gt}*ratio}{2},{b}_{b}^{gt}={y}_{c}^{gt}-\frac{{h}^{gt}*ratio}{2}$$

13 $${b}_{l}={x}_{c}-\frac{w*ratio}{2},{b}_{r}={x}_{c}-\frac{w*ratio}{2}$$

14 $${b}_{t}={y}_{c}-\frac{h*ratio}{2},{b}_{b}={y}_{c}-\frac{h*ratio}{2}$$

**Figure 5:**
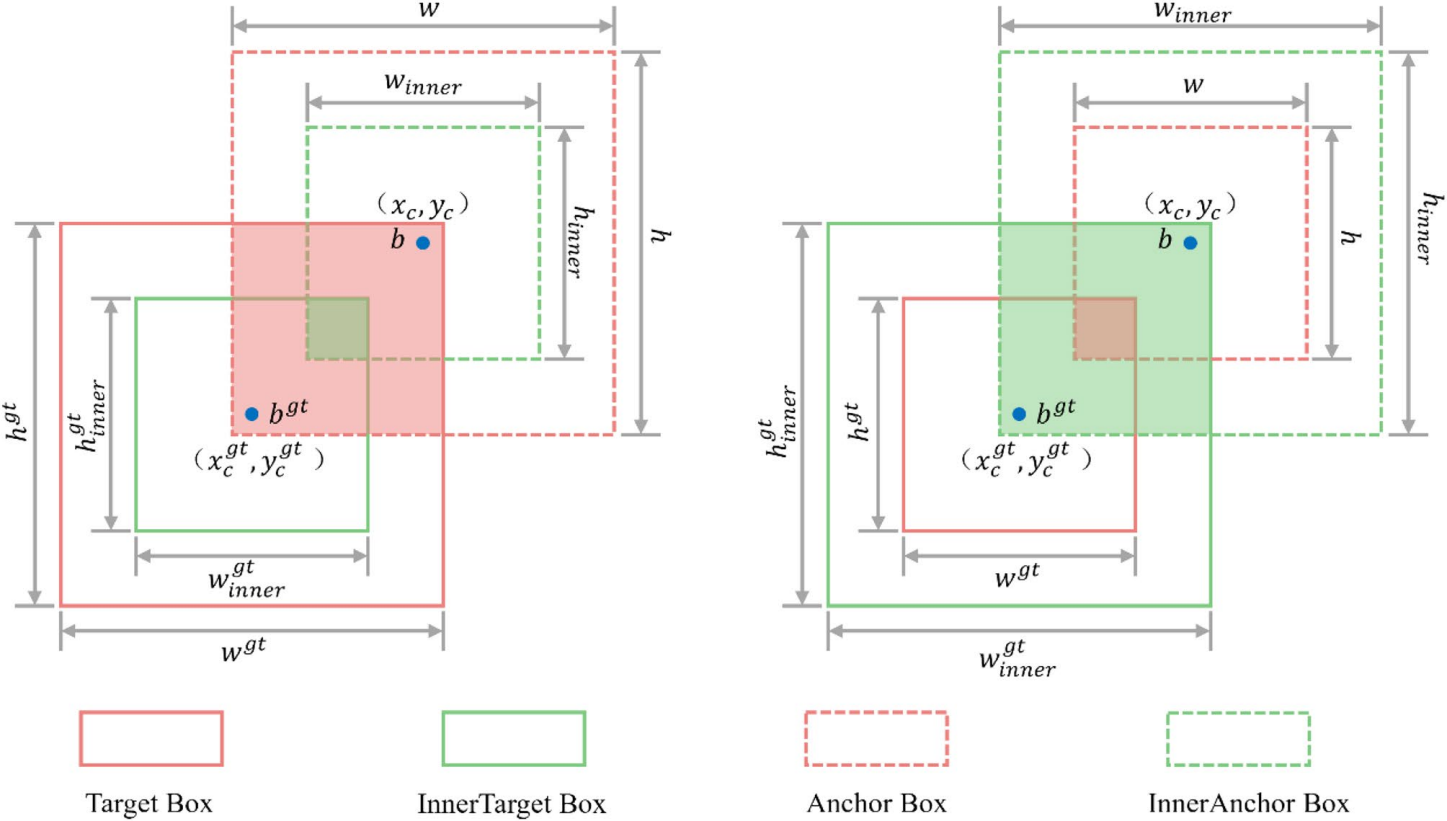
Auxiliary bounding box.

As shown in Fig. 5, ${x}_{c}$ and ${y}_{c}$ denote the coordinates of the centroid of the prediction box, ${x}_{c}^{gt}$ and ${y}_{c}^{gt}$ denote the coordinates of the centroid of the true box, $w$ and $h$ denote the width and height of the prediction box, ${w}^{gt}$ and ${h}^{gt}$ denote the width and height of the true box, and $ratio$ denotes the scaling factor for generating the auxiliary bounding box, which generally takes a range of values between [0.5, 1.5].

## Experimental results and analysis

### Experimental environment and model parameters

#### Experimental environment

The operating system used in this experiment is Windows 11 64-bit operating system, the CPU is Intel(R) Core(TM) i5-13400F @ 2.60 GHz, the GPU is NVIDIA GeForce RTX 3060 with 12 GB of video memory, the running memory is 16 GB, the programming language is Python 3.8, the deep learning framework is Pytorch 1.8.1, and CUDA version is CUDA 11.1.

#### Model parameters setting

In the training process of the model, the input image size is 608 × 608, the batch size is 8, the number of training rounds is 200, the optimizer is Adam optimizer, the momentum is 0.937, the weights decay is 0.0005, the initial learning rate is 0.01, and the learning rate is reduced by a cosine function.

##### Dataset preprocessing

**Figure 6:**
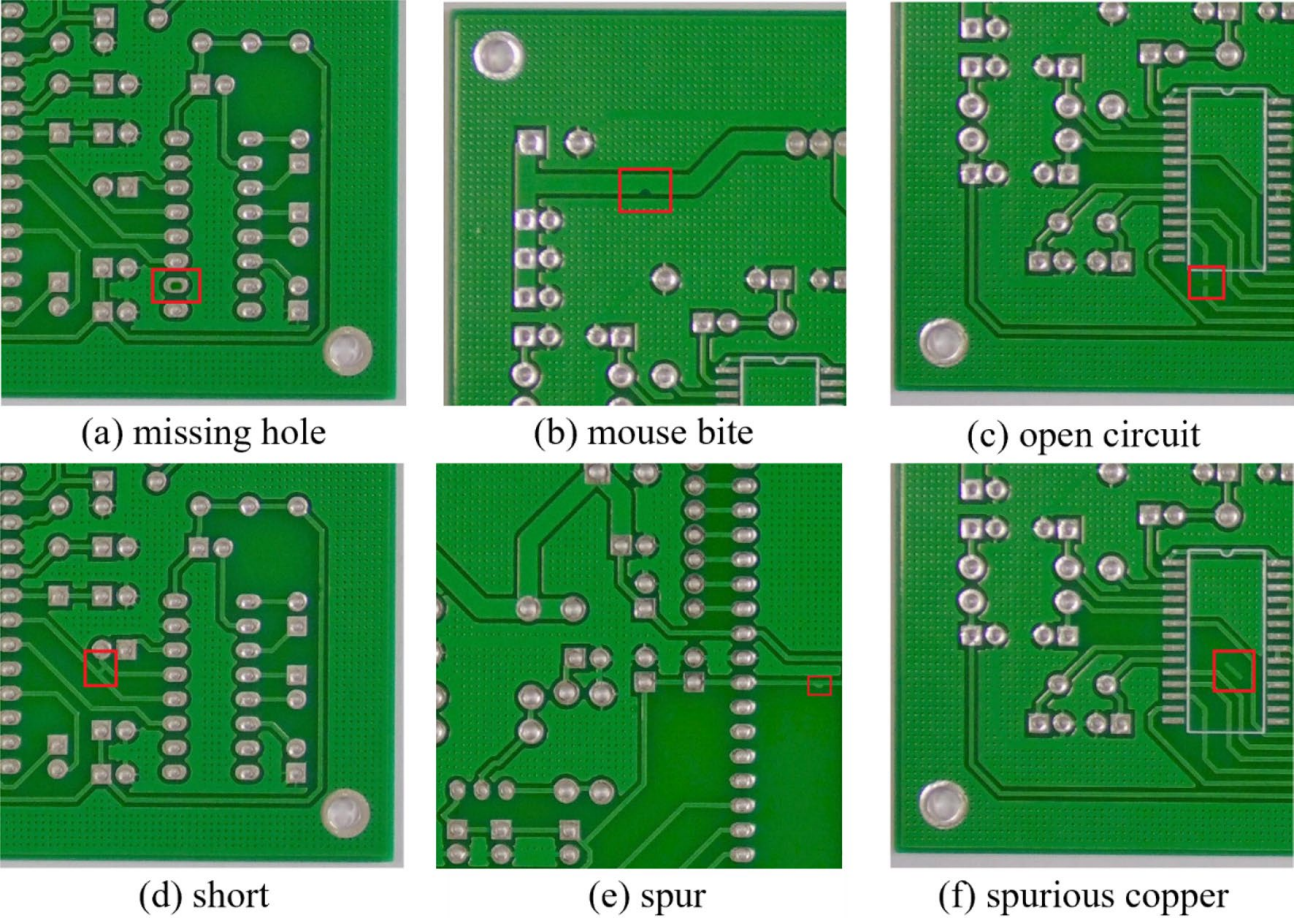
PCB defects.

The experiment utilised the PCB Defect dataset, released by the Intelligent Robot Open Laboratory of Peking University (http://robotics.pkusz.edu.cn/resources/dataset/). The dataset comprises 693 images of PCB defects, which were cropped to produce 10,668 images. The dataset consists of 10,668 images, each containing one of six types of defects: missing hole, mouse bite, open circuit, short, spur, and spurious copper. Table 1 shows the number of images for each defect type. The defects in the PCB images were labeled using the LabelImg tool and stored in the Pascal VOC dataset format. The dataset was then divided into a training set and a test set in an 8:2 ratio. Figure 6 displays images of defects in the PCB Defect dataset. The red boxed areas indicate the defective parts.

**Table 1:** Dataset of PCB defect.

Type of defects	Number of original images	Number of expanded images
Missing hole	115	1832
Mouse bite	115	1852
Open circuit	116	1740
Short	116	1732
Spur	115	1752
Spurious Copper	116	1760
Total	693	10,668

### Evaluation metrics

We use mean Average Precision (mAP), Parameters, GFLOPs, and FPS as evaluation metrics. mAP is the average of the AP values of different PCB defects, defined as in Eq. (15).

15 $$mAP=\frac{\sum_{i=1}^{N}{AP}_{i}}{N}$$

where N denotes the number of PCB defect types and AP is the area enclosed by the PR curve, the calculation formula is shown in Eq. (16).

16 $$AP={\int }_{0}^{1}P\left(R\right)dR$$

P is the precision, which indicates the probability of being correctly classified in the predicted positive sample and is calculated as shown in Eq. (17).

17 $$P=\frac{TP}{TP+FP}$$

where TP denotes the number of samples that are predicted to be positive and true positive samples and FP denotes the number of samples that are predicted to be positive but true negative samples.

R is the Recall, which represents the probability of being correctly classified among all positive samples and is calculated as shown in Eq. (18).

18 $$R=\frac{TP}{TP+FN}$$

where FN denotes the number of samples that are predicted to be negative but true to be positive.

### Analysis of results

#### Ratio setting of inner-CIoU loss

To determine the appropriate ratio, we conducted experiments using the CDI-YOLO network model on the PCB Defect dataset by setting the ratio of Inner-CIoU to 0.6, 0.7, 0.8, 0.9, and 1.1, respectively. Table 2 shows the results of the comparative experiments conducted on the PCB Defect dataset.

**Table 2:** Performance comparison of inner-CIoU with different ratios.

Ratio	P (%)	R (%)	mAP_50_ (%)	mAP_50:95_ (%)
0.6	96.3	93.0	96.6	47.7
0.7	**97.1**	**96.4**	**98.3**	**51.1**
0.8	**97.1**	95.6	97.9	51.0
0.9	96.1	93.6	97.2	49.2
1.1	95.4	92.5	96.2	47.6

By observing the experimental results in Table 2, we can see that when the ratio parameter is set to 0.7, the optimal values of P, R, mAP_50,_ and mAP_50:95_ are obtained. Based on this observation, we decided to set the ratio of Inner-CIoU to 0.7.

#### Ablation experiment

To evaluate the impact of the CA module, DSConv, and Inner-CIoU loss functions on the performance of the YOLOv7 tiny network model, we performed comparative experiments on the PCB Defect dataset. Table 3 shows the results of the removal experiments. In the table, we use Model_1 to denote the baseline YOLOv7-tiny model, Model_2 to denote the introduction of the CA module, Model_3 to denote the introduction of DSConv, Model_4 to denote the introduction of the Inner-CIoU loss function, Model_5 to denote the simultaneous introduction of the CA module and DSConv, and Model_6 to denote the simultaneous introduction of the CA module and the Inner-CIoU loss function, Model_7 to denote the simultaneous introduction of DSConv and the Inner-CIoU loss function, and Model_8 to denote the CDI-YOLO network model. The results of these ablation experiments allow us to evaluate the impact of each module on the model performance.

**Table 3:** Ablation experiment.

Model	AP_50_ (%)						mAP_50_ (%)	Parameters (M)	GFLOPs	FPS
	Missing hole	Mouse bite	Open circuit	Short	Spur	Spurious copper				
Model_1	98.7	95.0	94.3	95.9	95.1	93.0	95.4	5.74	13.1	131
Model_2	98.4	96.8	96.1	96.9	95.9	94.6	96.4	5.76	13.1	111
Model_3	98.5	96.4	95.8	95.9	95.3	95.4	96.2	5.74	12.5	139
Model_4	98.7	97.6	96.2	96.0	97.1	96.2	97.0	5.74	13.1	135
Model_5	**99.0**	97.0	95.6	97.3	95.5	96.2	96.8	5.76	12.6	120
Model_6	98.4	97.5	97.0	97.3	97.7	97.7	97.6	5.76	13.1	115
Model_7	98.5	97.5	96.3	96.9	97.6	97.6	97.4	5.74	12.5	140
Model_8	98.9	**97.8**	**98.3**	**97.6**	**98.3**	**98.6**	**98.3**	5.76	12.6	128

Significant values are in bold

From the experimental results of Model_2, it can be seen that the introduction of the CA module improves the feature extraction capability of the model for PCB defects and increases the mAP_50_ to 96.4. Compared to YOLOv7-tiny, an improvement of 1% is achieved. However, this improvement is accompanied by a small increase in the number of model parameters and a decrease in FPS. This is because when we introduce the CA module, we need to introduce coordinate encoding parameters to represent position information, and these parameters increase the number of model parameters. In addition, the CA module needs to operate on each position in the feature map during the computation process, which increases the computational complexity of the model and leads to an increase in the computation time for each forward propagation step, which decreases the FPS. As can be seen from the experimental results of Model_3, we can observe that after replacing some of the ordinary convolutions of YOLOv7-tiny with DSConv, the GFLOPs of the model decreased by 4.5%, while the FPS increased by 6.1%. This shows that DSConv can reduce the computational complexity of the model and increase the speed of recognition. With the experimental results of Model_4, we can see that after using Inner-CIoU as the loss function of YOLOv7-tiny, the convergence speed of the model is improved, the bounding box prediction is more accurate, and the detection accuracy and speed are also improved, making the model’s mAP_50_ reach 97.0%.

In the experimental results of Model_5, we observe that after the introduction of both the CA module and DSConv, DSConv can compensate to some extent for the decrease in FPS caused by the introduction of the CA module and reduce the model’s GFLOPs. The experimental results of Model_6 show that under the combined effect of the CA module and the effect of the Inner-CIoU loss function, the model’s mAP_50_ improves by 2.2%. Similarly, the experimental results of Model_7 show that with the combined effect of the DSConv and Inner-CIoU loss functions, the model’s mAP_50_ improves by 2.0% and FPS improves by 6.9%. Finally, in the experimental results of Model_8, we can see that after the simultaneous introduction of these three modules, the model’s mAP_50_ reaches 98.3%, which is the best performance among all the models. Model_8 has the best performance in terms of AP_50_ for the six PCB defect types, except that the AP_50_ of the missing hole is lower than that of Model_5, and the other five PCB defect types have the AP_50_ are all the highest.

In summary, compared to YOLOv7-tiny, CDI-YOLO performs well in all performance metrics except for a slight increase in the number of parameters and lower FPS.

#### Comparison experiment

To validate the advantages of the CDI-YOLO network model, we compared its performance with the existing mainstream methods (YOLOv3, YOLOv3-SPP, YOLOv4, YOLOR, YOLOv5s, YOLOv7, and YOLOv7-tiny) on the PCB Defect dataset. The results of the comparison experiments are shown in Table 4.

**Table 4:** Comparison experiment.

Model	P (%)	R (%)	mAP_50_ (%)	mAP_50:95_ (%)	Parameters (M)	GFLOPs	FPS
YOLOv3	91.1	89.5	94.1	46.6	58.7	154.6	57
YOLOv3-SPP	91.3	90.5	95.0	46.8	59.7	155.5	56
YOLOv4	92.4	91.2	95.8	47.5	44.3	114.1	60
YOLOR	93.2	90.1	96.1	47.7	44.3	114.1	58
YOLOv5s	92.7	91.0	95.9	47.3	6.73	16.3	115
YOLOv7	96.6	94.3	97.3	50.2	34.8	103.2	65
YOLOv7-tiny	95.2	90.4	95.4	46.9	**5.74**	13.1	**131**
CDI-YOLO	**97.1**	**96.4**	**98.3**	**51.1**	5.76	**12.6**	128

Significant values are in bold

Table 4 compares the performance of the different models in terms of P, R, mAP_50_, mAP_50:95_, Parameters, GFLOPs, and FPS. CDI-YOLO achieves the best results in terms of P, R, mAP_50_, MAP_50:95_, and GFLOPs. CDI-YOLO’s results are slightly worse than YOLOv7-tiny only in terms of Parameters and FPS.

In general, compared with the existing mainstream methods, CDI-YOLO is slightly inferior to YOLOv7-tiny in terms of the number of parameters and detection speed, but the gap is not large. It is worth noting that CDI-YOLO shows higher detection accuracy on the PCB Defect dataset. This proves that CDI-YOLO can solve the problems that the existing methods cannot achieve at the same time in terms of high detection accuracy, fast detection, and fewer parameters, making it a suitable choice for real-time detection deployed on hardware devices.

#### Results of the test

**Figure 7:**
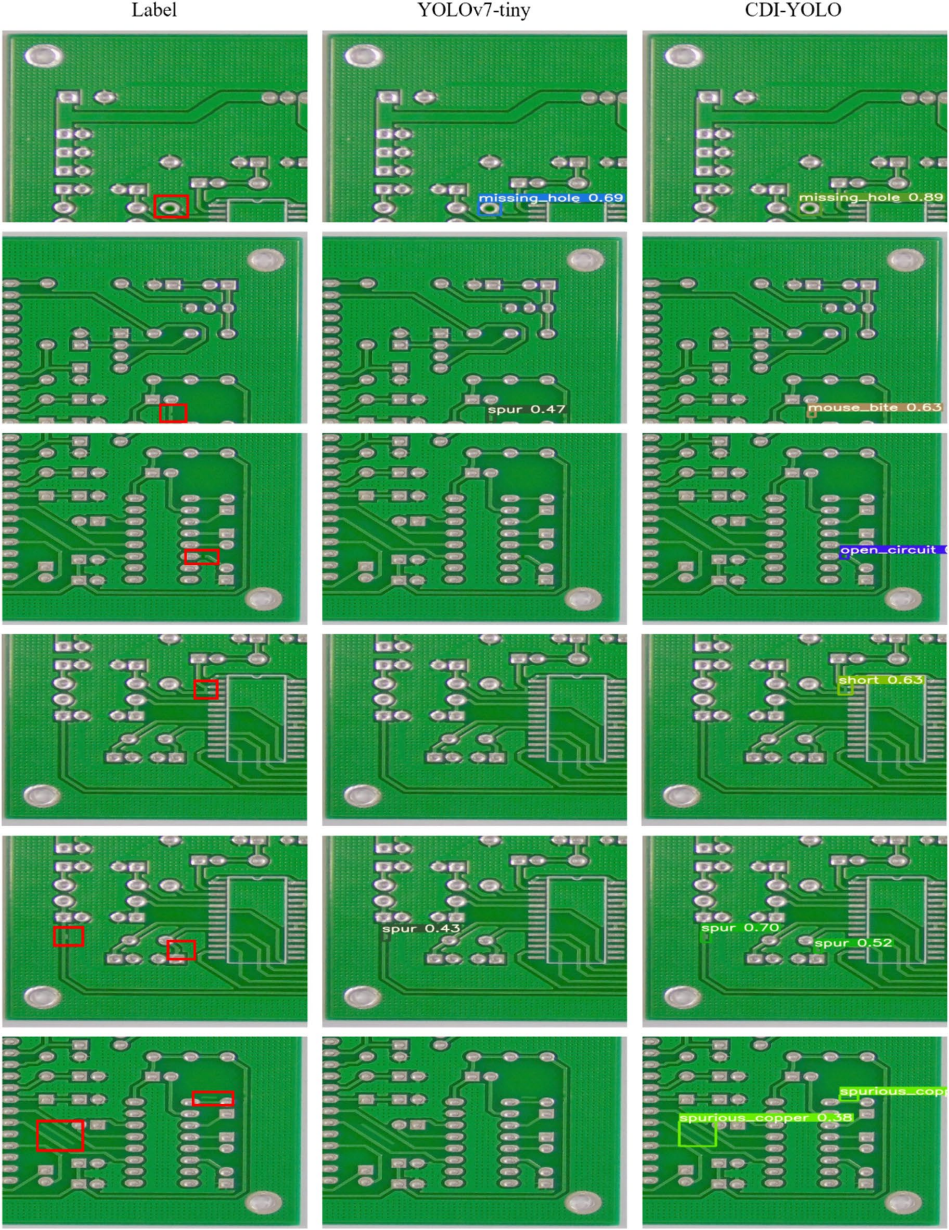
Comparison of the detection results of YOLOv7-tiny and CDI-YOLO.

Figure 7 shows a comparison of the detection results of the YOLOv7-tiny algorithm and the CDI-YOLO algorithm on six types of PCB defects. The first column shows the location of the defect with a red box, the second column shows the detection results of the YOLOv7-tiny algorithm, and the third column shows the detection results of the CDI-YOLO algorithm. Each row uses the same image and the corresponding defect types are missing hole, mouse bite, open circuit, short, spur, and spurious copper.

Looking at the detection results in row 1, we can see that the YOLOv7-tiny algorithm has a lower confidence level in its detection results compared to the CDI-YOLO algorithm. In the detection results of row 2, the YOLOv7-tiny algorithm has a false detection situation. In the detection results of rows 3, 4, 5, and 6, the YOLOv7-tiny algorithm has a missed detection situation. Taken together, the detection results of the CDI-YOLO algorithm are better than those of the YOLOv7-tiny algorithm.

## Conclusion

This paper proposes a PCB defect detection algorithm based on CDI-YOLO. The algorithm introduces a CA module into the YOLOv7-tiny object detection algorithm to better understand and utilize spatial information, thereby enhancing perception and reasoning capabilities for detecting defects at different positions on the PCB. Additionally, selected regular convolutional layers are replaced with DSConv to reduce model complexity and improve detection speed. Furthermore, Inner-CIoU is employed as the bounding box regression loss function, leveraging auxiliary bounding boxes to expedite the model’s bounding box regression speed. Experimental results demonstrate that CDI-YOLO achieves the highest mAP of 98.3% in terms of detection accuracy compared to existing methods. In terms of parameters, CDI-YOLO has 5.8 M parameters, slightly higher than YOLOv7-tiny but with negligible difference. In terms of detection speed, CDI-YOLO achieves a speed of 128 FPS, slightly lower than YOLOv7-tiny but capable of meeting real-time detection requirements. Therefore, the proposed method successfully addresses the simultaneous challenges of achieving high detection accuracy, fast detection, and reduced parameter count, providing an excellent solution for practical PCB defect detection systems.

However, in practical application scenarios, there are various interfering factors such as complex backgrounds, lighting variations, and noise, which can affect the accuracy of the model’s detection. To improve the detection accuracy of the model in real-world scenarios, we plan to augment our dataset of PCB defect samples with more instances that contain complex backgrounds. This will allow us to train our model and enhance its robustness. Additionally, annotating a large number of defect samples in PCB defect detection is a time-consuming and expensive task. Future research can explore the use of weakly supervised learning methods, such as weak labeling, unlabeled data, and semi-supervised learning, to improve the effectiveness of defect detection. This approach will help reduce the demand for a large amount of annotated data, thereby lowering costs and improving detection performance.

## Data Availability

The datasets analysed during the current study are available in the https://robotics.pkusz.edu.cn/resources/dataset/.
